# Treatment of Inflammatory Dentigerous Cyst Using a Surgical T Drain in a Child

**DOI:** 10.1055/s-0042-1756688

**Published:** 2022-10-11

**Authors:** Antonella Lešin, Ivan Galić, Antonija Tadin, Katarina Vilović, Daniel Jerković

**Affiliations:** 1 Department of Maxillofacial Surgery, University Hospital of Split, Split, Croatia; 2 Department of Maxillofacial Surgery, University Hospital of Split, School of Medicine, University of Split, Split, Croatia; 3 Department of Restorative Dental Medicine and Endodontics, School of Medicine, University of Split, Split, Croatia; 4 Department of Anatomy, Department of Pathology, University Hospital of Split, School of Medicine, University of Split, Split, Croatia

**Keywords:** dentigerous cyst, decompression, T drain, children, oral surgery

## Abstract

Dentigerous cysts are rarely reported in young children. They are usually asymptomatic and only identified when becoming significantly large. Treatment by enucleation may damage structures like the inferior alveolar nerve, maxillary sinus, or permanent teeth, thus reducing the child's quality of life. Therefore, conservative surgical treatment such as decompression is indicated. This case report describes the treatment and subsequent complete regression of an inflammatory dentigerous cyst based on the decompression method using a customized surgical tube in a 10-year-old girl. The innervation was preserved, and permanent teeth erupted.

## Introduction

The dentigerous cyst (DC), also called a follicular cyst, is odontogenic in nature and includes the crown of an unerupted or impacted tooth. 1 2 Though it is the second most common jaw cyst affecting 0.9 to 7.3% of the population, dental literature reports a low prevalence in children. 2 3 The condition is most often found in persons aged in their thirties. Only 4 to 9% of all DCs occur in the first decade of life. 4 5 6

Their origin can be developmental or inflammatory, but their exact etiology remains unclear. An inflammatory dentigerous cyst (IDC) appears around an unerupted permanent tooth due to inflammation spreading from an overlying nonvital primary tooth. 7 It occurs most often in the mandibular premolar region, where primary molars are damaged by caries. 5 8

Smaller DCs are generally asymptomatic and accidentally discovered, for instance, during a routine radiographic examination. Larger cysts may cause expansion of the bone resulting in facial asymmetry, root resorption, and shifting of adjacent teeth. 8

A follicular cyst radiographically appears as a well-defined unilocular radiolucency surrounding the crown of an unerupted tooth. Inflammatory types usually involve the roots of a nonvital primary tooth and the crown of an unerupted permanent successor that can be displaced. 7 8

A correct diagnosis requires histopathological analysis because unicystic ameloblastoma and odontogenic keratocysts exhibit similar radiographic features. 8

The DC is treated using enucleation, marsupialization/decompression, or a combination of the two procedures. Enucleation should be done for any cyst that can be safely removed without sacrificing adjacent structures. 9 10 However, when treating larger cysts, or those present in pediatric patients with mixed dentition, the decompression method is preferred as it protects the unerupted permanent successors. 11

## Case Report

A 10-year-old girl was referred to the Department of Maxillofacial and Oral Surgery for painless swelling on the right side of the mandible. Intraoral examination revealed a normal-looking mucosa with a thin expansion of the buccal cortical, exhibiting bone elasticity on palpation in the primary mandibular right first molar region. The patient denied any sensory deficit. There was no account of specific systematic diseases or previous traumatic injuries in the affected area.

**Fig. 1:**
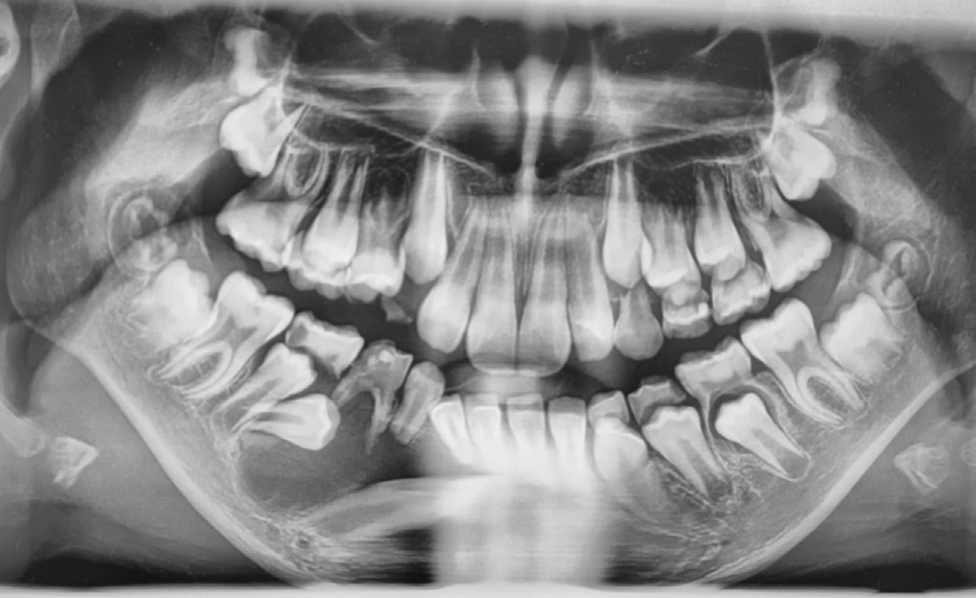
Preoperative panoramic radiograph of a 10-year-old girl showing unicystic radiolucency on the right side of the mandible with an unerupted permanent canine and premolars.

**Fig. 2:**
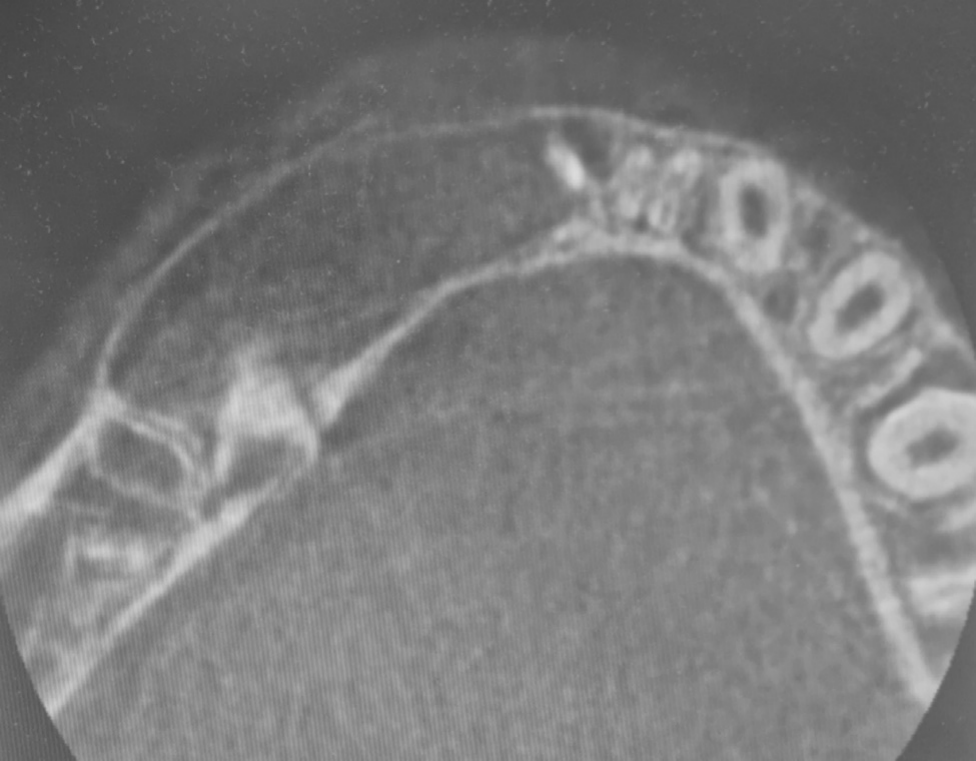
Preoperative cone-beam computed tomography in the axial plane of the lesion measuring 4.1 × 2.4 cm in size. Expansion of the vestibular cortical bone was also observed.

A panoramic radiograph and a cone-beam computed tomography (CBCT) brought in by the mother showed significant unicystic radiolucency, with well-defined margins expanding from the primary mandibular right second molar to the permanent central incisor on the same side. The nonerupted permanent canine was horizontally shifted, and the first premolar mesially inclined, while the second premolar seemed to be typically positioned. The roots of the central and lateral right incisors were tilted, and the inferior alveolar nerve was in contact with the lesion. The primary first molar was significantly damaged by caries and nonvital. The root of the primary canine was resorbed ( Figs. 1 and 2 ).

Based on these clinical and radiological findings, a provisional diagnosis of an IDC caused by the primary mandibular right first molar was made.

**Fig. 3:**
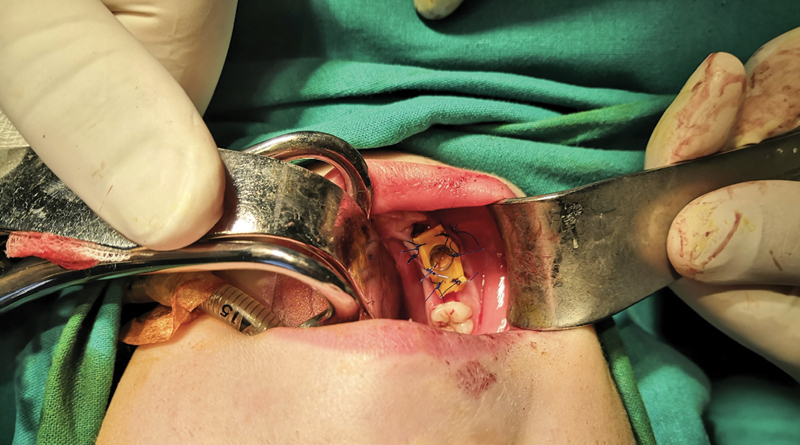
A customized decompression device set in place and secured with sutures. The tube entrance has a satisfactory width, ensuring easy application of the cannula for irrigation.

The primary mandibular right canine, including the first and second molars, were extracted under general anesthesia due to the patient's age and fear of the procedure. First, an incisional biopsy for the histopathological examination was performed. Then, a decompression device made from a prefabricated surgical T drainage tube (T-FR Huali Technology No.666 Chaoqun street High tech area, Changchun, Jilin, China) was used. It was cut precisely to the desired length and width from measurements on a preoperative CBCT, and its vertical end was positioned inside the cystic lumen. Next, the horizontal part (wings) was drilled on both sides, providing an easier fixation on the mucosa. The device was inserted into the extraction socket of the primary first molar and secured with 4–0 nylon sutures ( Fig. 3 ).

The patient's parents were instructed to irrigate the cyst cavity using 10 mL syringes filled with 0.9% saline solution by inserting the plastic part of the cannula into the tube entrance three times a day. Postoperative follow-up appointments were scheduled to take place every 3 months.

**Fig. 4:**
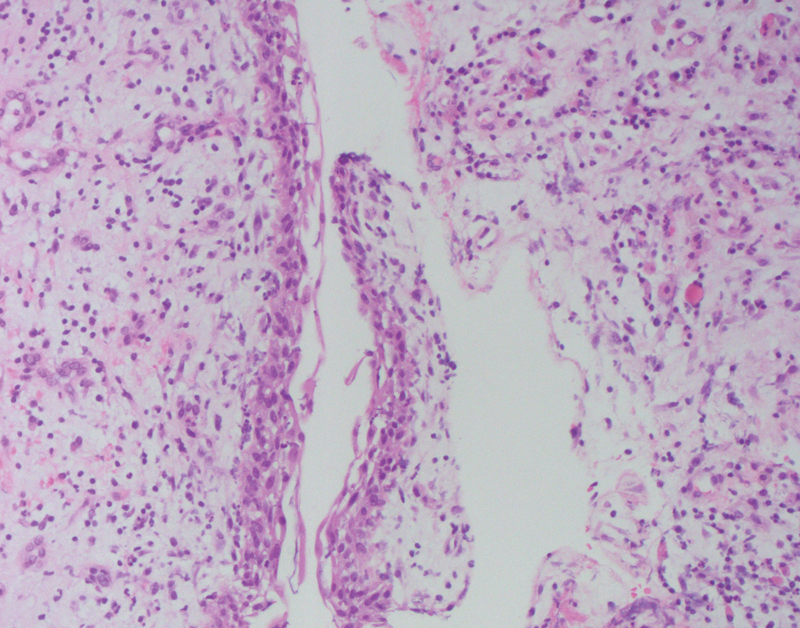
Dentigerous cyst, hematoxylin and eosin, 200x. Histology revealed an inflamed wall of fibrous tissue lined with four layers of squamous epithelium.

A histopathological examination of the lesion confirmed the clinical diagnosis of the IDC ( Fig. 4 ).

**Fig. 5:**
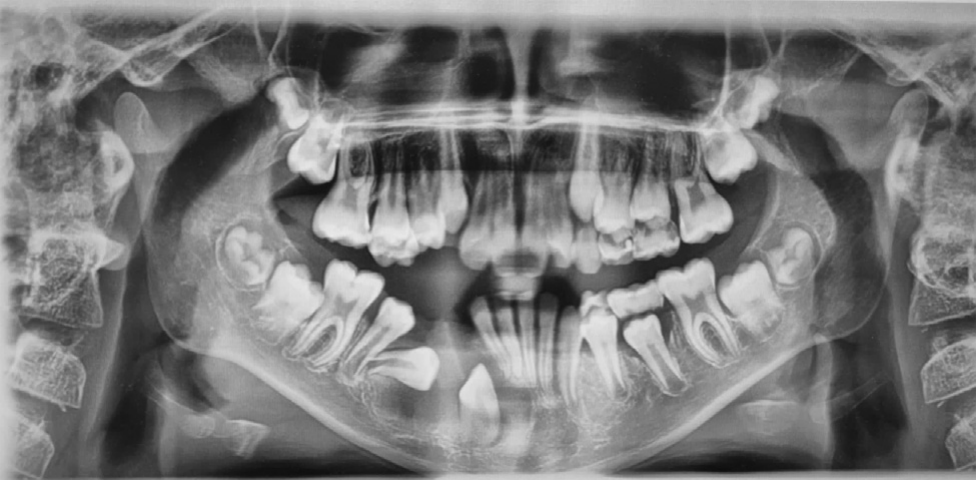
Three-month postoperative radiograph showing reduced radiolucency and eruption of the second premolar with a more vertical canine position.

**Fig. 6:**
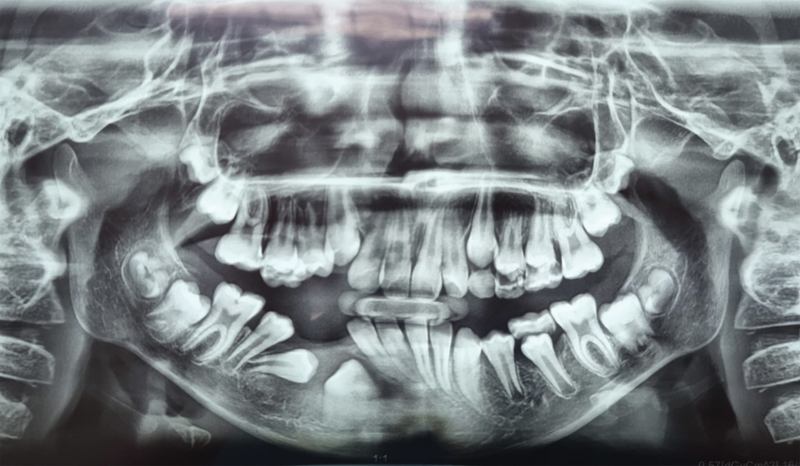
Six-month postoperative radiograph showing further reduction of the lesion. Eruption of the second premolar was observed, and the decompression tube was removed.

Three months later, the postoperative radiograph showed a more vertically positioned canine with reduced radiolucency ( Fig. 5 ). The decompression tube needed to be shortened due to the canine eruption. A significant lesion regression was observed in the 6-month follow-up, leading to the removal of the drain ( Fig. 6 ).

**Fig. 7:**
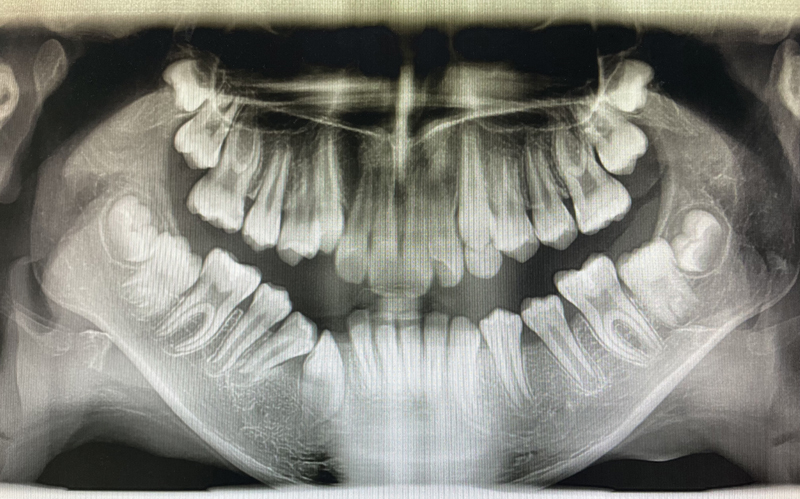
Twelve-month postoperative radiograph showing the spontaneous eruption of all permanent teeth and lesion resolvement.

A year after the decompression had been done, all permanent teeth involved in the eruption process maintained vitality. The complete regression of the lesion with bone formation was radiographically observed ( Fig. 7 ), and the innervation of the right inferior alveolar nerve was preserved entirely. The patient was referred to an orthodontist to correct the rotated canine position.

## Discussion

Even though the histopathology of the follicular cyst remains unclear, its connection to inflammation caused by the nonvital primary tooth is obvious. A study involving a histological evaluation of cysts occurring in the mixed dentition stage detected an inflammatory process caused by a primary tooth in 93.6% of the observed follicular cysts. 12 Based on this information, removing the source of inflammation, that is, the primary mandibular right first molar in our patient, is the essential therapeutic procedure.

Several authors have shown that decompression is an effective treatment for odontogenic cysts. 13 14 It is a conservative technique that retains the permanent teeth, pulp vitality, and in this case, essential structures like the inferior alveolar nerve. However, this approach requires compliance from the patient. 14

Reducing intraluminal pressure and facilitating bone formation requires keeping the cyst open. This is done using various devices, such as a simple iodoform gauze, stents, brackets and chains attached to impacted teeth, or using removable partial dentures that act like obturators. 15 16 In our case, we used a tube modified from a surgical T drain and secured with sutures. It was practical given that the material is soft and does not damage the underlying mucosa. Also, it can be easily cut to the desired length, and its “wings” helped keep it from accidentally moving into the bone defect. Even though tube maintenance can be challenging for patients, especially children, the patient's mother said it became part of their daily routine. Besides some adjustments performed during the checkup appointments, we did not observe commonly reported problems like infection or obliteration of its entrance. 17

Full eruption of the involved permanent teeth and healing of the cystic cavity in our patient occurred after 12 months, which is somewhat longer than Allon et al reported, where the estimated mean decompression period is 7.5 months in children under 18 years of age. 18 This outcome may be due to the lesion's size or the case's specifics.

Previous case reports, as well as ours, show that the permanent successors, even when badly dislocated, erupt into the dental arch. 19 A systematic review by Nahajowski et al showed that a patient's young age (∼10 years) and root formation below half its total length seem to be factors that increase the probability of a spontaneous eruption. 20

Not many published studies report DCs treated using decompression in children, which may be due to the low incidence of DCs in that population. Therefore, further studies of this kind should be conducted.

## Conclusion

IDCs can be treated successfully with minimal intervention using a conservative method like decompression. By extracting the infected primary teeth and ensuring continuous drainage utilizing a device like ours, essential structures can be protected and spontaneous eruption of the permanent teeth achieved, thus reducing the need for prosthetic rehabilitation. The patient should be scheduled for regular follow-ups until the healing process has been completed.
